# Challenges and Strategies in the Synthesis of Mesoporous Alumina Powders and Hierarchical Alumina Monoliths

**DOI:** 10.3390/ma5020336

**Published:** 2012-02-20

**Authors:** Sarah Hartmann, Alexander Sachse, Anne Galarneau

**Affiliations:** Institut Charles Gerhardt Montpellier, UMR 5253 CNRS/UM2/ENSCM/UM1, ENSCM 8 Rue de l’Ecole Normale, 34296 Montpellier Cedex 5, France; E-Mails: s.j.hartmann@web.de (S.H.); alexandersachse82@gmail.com (A.S.)

**Keywords:** mesoporous alumina, high surface area alumina, alumina monolith, hierarchical porosity, nanostructured alumina, catalyst support

## Abstract

A new rapid, very simple and one-step sol-gel strategy for the large-scale preparation of highly porous γ-Al_2_O_3_ is presented. The resulting mesoporous alumina materials feature high surface areas (400 m^2^ g^−1^), large pore volumes (0.8 mL g^−1^) and the γ-Al_2_O_3_ phase is obtained at low temperature (500 °C). The main advantages and drawbacks of different preparations of mesoporous alumina materials exhibiting high specific surface areas and large pore volumes such as surfactant-nanostructured alumina, sol-gel methods and hierarchically macro-/mesoporous alumina monoliths have been analyzed and compared. The most reproducible synthesis of mesoporous alumina are given. Evaporation-Induced Self-Assembly (EISA) is the sole method to lead to nanostructured mesoporous alumina by direct templating, but it is a difficult method to scale-up. Alumina featuring macro- and mesoporosity in monolithic shape is a very promising material for in flow applications; an optimized synthesis is described.

## 1. Introduction

The industrial interest of alumina is highlighted by its intensive use as catalyst or as catalytic support materials for the petroleum refinement and as automobile emission controller [1,2]. The use of alumina can be ascribed to both high thermal stability and moderate Lewis acidity as well as to the fact it is a rather inexpensive material. γ-Al_2_O_3_ is commonly used as support for nanocrystal of MoS_2_ doped with Co or Ni for hydrodesulfurization (HDS) processes [3]. Recently, high surface area nanostructured alumina has allowed to increase the catalytic behavior of HDS catalysts [4]. Therefore it is important to develop strategies to design simple, reproducible and easy to scale-up procedures leading to high surface area, high pore volume and high pore size alumina by simple methods to be implemented in industrial processes.

The introduction of defined porosity within bulk alumina is requested for many other catalytic applications besides oil refining and sorption applications [5,6]. This is mainly due to the fact that the presence of mesopores enhances the diffusion properties of the material and increases the amount of accessible active sites [7]. The preparation of porous alumina with well-defined mesopores and high surface areas has been reported by several groups by the use of surfactant-directing agents [8,9]. The group of Zhao recently extended the synthesis of nanostructured mesoporous alumina by the evaporation-induced self-assembly (EISA) with tailorable structure and pore size assisted by the organic swelling agent trimethylbenzene [10]. Furthermore, the nanocasting method gives a large spectrum of possible mesoporous aluminas by the diversity of hard templates available [11]. These synthetic routes are rather time consuming as it uses a double replica route through a carbon phase and rely on multi-step synthesis and are thus difficult to scale up. For many industrial applications very narrow pore size distributions are not necessary. Several attempts in the literature describe the synthesis of porous alumina with wide size distribution of mesopores [12]. The group of Satcher developed a simple synthetic route to mesoporous alumina by the use of epoxides as gelation initiators in the preparation of sol-gel materials [13]. Yao *et al.* have fabricated amorphous alumina by a sol-gel process using ultrasound to provide energy [14].

The preparation of aluminas with hierarchical porosity with meso- and macropores have attracted a great deal of attention for catalytic applications, as pores on different length scales can assume diverse functions such as high dispersion of active site and high mass transfer [15]. Dacquin *et al.* [16] have for example shown the fabrication of macro-mesoporous alumina by combining surfactant and latex spheres (300–400 nm) templating methods. However the macropore sizes in combination with a delicate alumina skeleton seem to limit their applicability as stationary phases in catalysis in flow, as the macropore size reduces the mass transfer within the monolith and the backbone probably can not withstand the resulting back pressure. Hierarchical systems such as macro and mesoporous monoliths with large macropores (3 microns) based on silica or alumina-lined silica have recently shown their potentiality as catalytic flow microreactors for the fine chemical production with low pressure drop (<0.5 bars) [17,18,19]. The morphological control of hierarchical alumina monoliths thus gives rise to a new class of promising candidates for application as catalytic microreactors.

The aim of this publication is hence to compare the synthetic routes of the literature and to propose the easiest and the most reproducible synthesis to produce mesoporous alumina with the desired properties. The selected synthetic routes present the formation of ordered and disordered mesoporous alumina powders featuring large mesopores, high surface areas and large pore volumes, as well as the synthetic strategies for the achievement of hierarchical alumina monoliths with large macropores efficient for continuous flow applications. Furthermore, a new synthetic route will be presented for the formation of mesoporous alumina by a very simple, one step sol-gel process resulting in a highly porous γ-Al_2_O_3_ phase obtained at low temperature.

## 2. Results and Discussion

### 2.1. Nanostructured Mesoporous Amorphous Al_2_O_3_ Powders

In 2008, Yuan *et al.* were among the first to present a facile synthesis procedure for the preparation of nanostructured mesoporous Al_2_O_3_ powders [20]. By employing amphiphilic block copolymers such as Pluronic^®^ P123 and F127 formed of ethylene-oxide (EO) and propyleneoxide (PO) groups (EO_20_PO_70_EO_20_ and EO_100_PO_65_EO_100_, respectively) in an evaporation-induced self-assembly process (EISA), highly ordered mesoporous organic-inorganic nanocomposites were obtained under controlled evaporation at 60 °C. Several groups have tried to use P123 as structuring agent for the nanostructuration of alumina [4,21,22], however without the use of EISA process no ordered alumina phases have been obtained.

**Figure 1:**
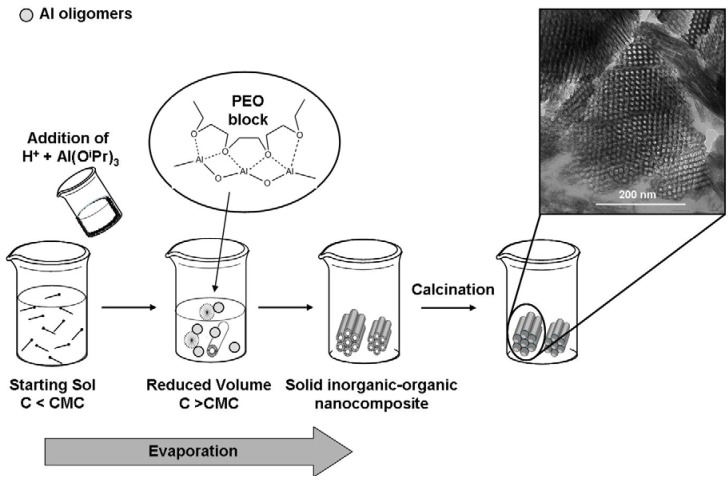
Schematic depiction of the formation of the nanostructured mesoporous alumina via the evaporation-induced self-assembly mechanism (EISA). The TEM image was taken from our own preparation (see experimental section) of an as-synthesized alumina material showing the 2D-hexagonally arrangement of mesopores (calcined at 400 °C for 4 h).

**Figure 2:**
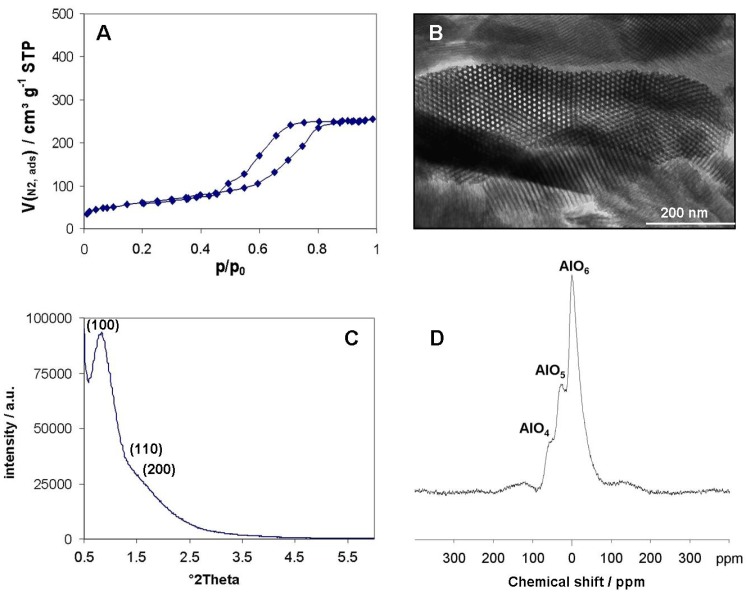
(**A**) Nitrogen sorption isotherm at 77 K of hexagonal mesoporous alumina with amorphous walls; (**B**) corresponding TEM image of the 2D-hexagonally arranged mesopore system; (**C**) Small X-Ray Diffraction recorded in the range showing the hexagonal arrangement; (**D**) ^27^Al MAS NMR spectra recorded at a spinning rate of 10 kHz.

Among the different proposed EISA processes [10,20,23,24], the one proposed by Yuan *et al.* [20] seems to be the simplest and the fastest to get mesoporous alumina powder. In this acidic EISA process, it is assumed that the multivalent hydrolysed and partly condensed Al species are preferentially assembled in the ethylene oxide blocks of the structure-directing co-polymer via weak coordination bonds [25]. As concentrated acids are employed as catalysts, this association is even enhanced by hydrogen bonding, allowing a systematic growth of the Al oligomers in the hydrophilic domains, which finally results in the formation of a mesoscopically ordered inorganic-organic nanocomposite. In the original publication of Yuan *et al.* [20], the recipe for the synthesis of nanostructured alumina is not clearly formulated, as a wide range of concentrations for each reactant and of acids is given. Other recipes for the EISA process have been formulated by the groups of Jaroniec and Wilson [26,27]. We successfully obtained a nanostructured mesoporous amorphous alumina (Figure 1) by following the recipe we present in the experimental section. It is important to avoid washing or liquid extraction before drying is completed, as the organic template prevents the mesoporous structure to collapse during the drying step. Only after a substantial drying at 60 °C leading to the solidification of the amorphous alumina phase, the calcination at 400 °C can be proceed and a free accessible 2D-hexagonally arranged mesopore system is achieved (Figure 2).

Thermogravimetric analysis confirmed that after calcination at 400 °C the structure-directing agents have been removed. Via this synthesis procedure nanostructured 2D-hexagonally mesoporous Al_2_O_3_ powders were synthesized featuring specific surface areas of 197 m² g^−1^, pore volume of 0.38 mL g^−1^ and mesopore diameter of 8.6 nm by employing EO_20_PO_30_EO_20_ as structure-directing agent and concentrated HNO_3_ as acidic catalyst (Figure 2A). Even though this synthesis was conducted in acidified ethanolic solution without addition of water, the water contents in nitric acid and in ethanol are sufficient to induce the hydrolysis of the Al(O*i*Pr)_3_ precursor. As can be seen from the X-ray diffraction pattern (Figure 2C) and the TEM image (Figure 2B), the 2D-hexagonal ordering of the mesopore system is highly evolved and XRD pattern shows a d-spacing d_100_ of 10.8 nm. The diffraction pattern in the wide-angle range indicates that after calcination at 400 °C the pore walls are completely amorphous. The corresponding ^27^Al MAS NMR spectra (Figure 2D) of the sample shows three resonance signals at 0, 29 and 62 ppm which can be related to the octahedral coordinated AlO_6_, the pentahedral coordinated AlO_5_ and the tetrahedral coordinated AlO_4_ sites in the alumina matrix. Penta-coordinated alumina is often associated to the presence of amorphous alumina phase, which is the case in this preparation. The penta-coordinated alumina disappeared in the benefit of hexa-coordinated alumina for a calcination at 900 °C giving the γ-Al_2_O_3_ phase. These results are in accordance with the literature [4,10,20] except that lower surface areas (200 instead of 300–400 m^2^ g^−1^) and pore volumes (0.4 instead of 0.8 mL g^−1^) have been obtained for a similar pore size.

Even if this synthesis is the more promising route to synthesized nanostructured mesoporous alumina, drawbacks have been discerned for this synthesis procedure that are: (1) smaller pore volumes and specific surface areas than expected (200 m^2^ g^−1^ and 0.4 mL g^−1^); (2) a large amount of organics to be eliminated as as-synthesized alumina features 70 wt% of organics, resulting in product yields below 30%; (3) the amorphous nature of the resulting alumina phase (high temperatures of >800 °C are necessary to obtain the γ-Al_2_O_3_ phase); and (4) difficulty to scale-up. Indeed for the development of this synthesis, the control of the EISA synthesis parameters such as humidity, temperature as well as the recipient volume to surface ratio in which the sol is evaporated are crucial, which makes it difficult to scale up the batch sizes for this procedure.

### 3.2. Disordered Mesoporous γ-Al_2_O_3_ Powders

If the desired application does not require a well-ordered mesoporous Al_2_O_3_ phase, as for theoretical study for instance, the synthesis of disordered mesoporous alumina materials based on sol-gel methods can be more convenient and efficient. In contrast to the previous synthesis that is difficult to scale-up via evaporation-induced self-assembly and requires an expensive structure-directing agent such as Pluronic P123, the synthesis of disordered mesoporous Al_2_O_3_ by careful sol-gel methods based on alumina hydrolysis control ([4] and references therein) leads to high surface area (~350 m^2^ g^−1^) and high pore volume (~0.8 mL g^−1^) mesoporous alumina which are much more easily scalable. Among the possible sol-gel methods, we have developed a new, one-step, easy and fast synthesis to obtain disordered mesoporous γ-Al_2_O_3_ at low temperature (500 °C) by using 2-butoxyethanol as solvent and as controlling agent for the Al(O*sec*Bu)_3_ hydrolysis and a small quantity of dodecylamine to speed up the synthesis leading to a rapid precipitate of alumina powder.

**Figure 3:**
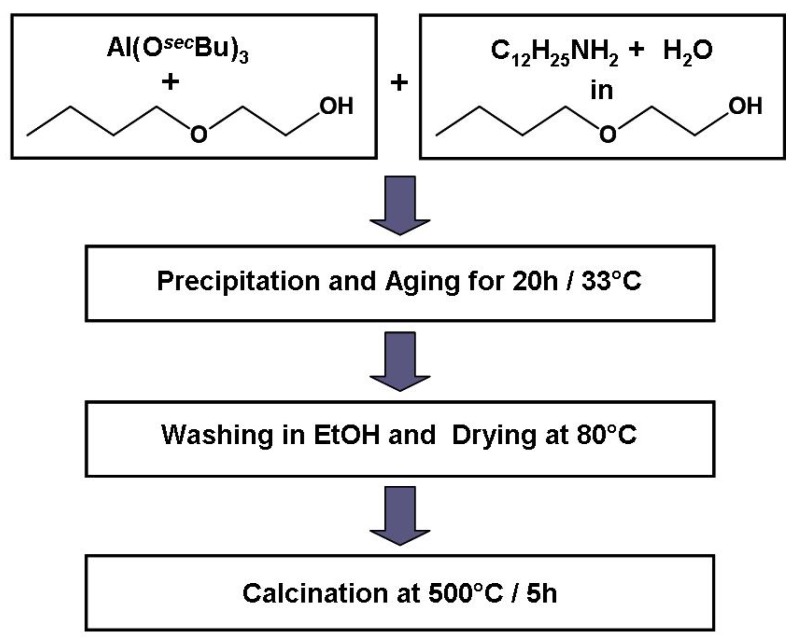
Synthesis route for the rapid preparation of disordered mesoporous γ-Al_2_O_3_.

**Figure 4:**
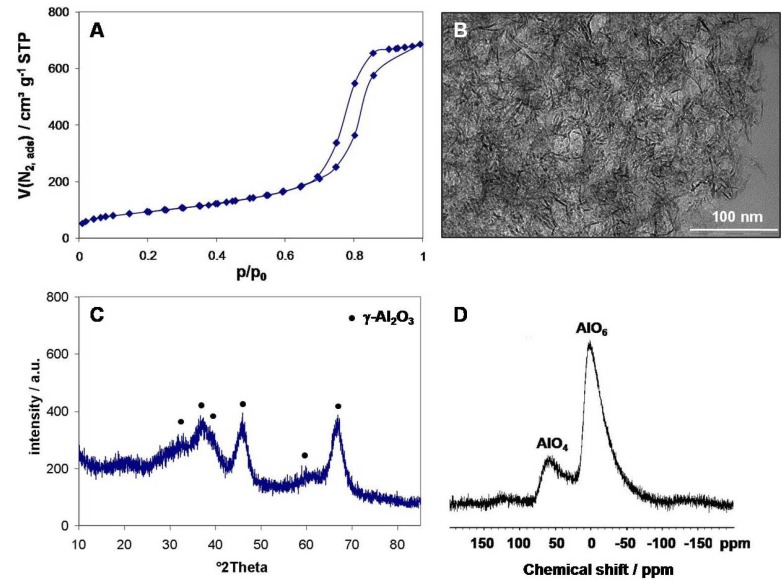
(**A**) Nitrogen sorption isotherms at 77 K of mesoporous disordered γ-Al_2_O_3_ phase calcined at 500 °C; (**B**) TEM image showing the fibrillar morphology of the material; (**C**) XRD pattern showing the peaks assigned to the γ-Al_2_O_3_ phase; (**D**) ^27^Al MAS NMR spectra for which penta-coordinated Al sites are absent as expected for a crystalline alumina phase.

The water and dodecylamine containing solution is added drop-wise within 20 min under vigorous stirring. With the addition of the first drops, hydrolysis and condensation reactions lead to an instant gelation of the system. The gel is only destroyed by further addition of the aqueous solution resulting in the precipitation of alumina which is then aged for 20 h at 33 °C. After washing in ethanol and drying at 80 °C the obtained powder consists of an amorphous alumina phase, which is then transformed into a semi-crystalline γ-Al_2_O_3_ phase by calcination at 500 °C for 5 h as can be seen from the XRD pattern in Figure 4.

This mesoporous alumina material combines its crystalline attributes of the γ-Al_2_O_3_ phase with high specific surface areas of 338 m^2^ g^−1^, high pore volumes of 1.1 mL g^−1^ and large mesopore sizes of 11 nm, which makes it a suitable candidate as inorganic support material for heterogeneous catalysis (Figure 4A). ^27^Al MAS NMR shows the sole presence of tetrahedral and octahedral coordinated aluminium species as expected for a crystalline alumina phase. This procedure not only has proven to be a highly reproducible synthesis but also features less organics to be treated, as only 30 wt % of organics are present in the as-synthesized “sol-gel” alumina resulting in a 70% yield with respect to mass.

### 3.3. Hierarchical Macro-/Mesoporous Al_2_O_3_ Monoliths

To perform catalysis under continuous flow condition, alumina should be obtained as monoliths with hierarchical pore structure as for silica [17,19]: large macropores (>1 µm) for high mass transfer with low drop pressure and high specific surface areas for a good dispersion of active sites. To our knowledge, the only procedures affording alumina with large macropore suitable for continuous flow applications have been proposed by the groups of Yuan and Nakanishi [28,29]. The synthesis developed in the Nakanishi group is based on a phase separation named spinodal decomposition between an alumina/polyethylene oxide (PEO) phase and an aqueous/PEO phase. In contrast to the previous sol-gel methods described for the syntheses of alumina powders, the preparation of alumina monoliths with hierarchical porosity (macro- and mesoporosity) demands a perfect control of parameters such as temperature, H_2_O content and pH. The exactitude of these parameters is crucial to control the hydrolysis and condensation reactions and to avoid precipitation. Based on this synthesis procedure [28], the employment of an acidic aluminium salt such as AlCl_3_·6H_2_O in ethanol/water mixture instead of an aluminium alkoxide precursor helps to avoid the rapid hydrolysis and condensation reactions due to formation of sole [Al(H_2_O)_6_]^3+^ at pH values below 3 [30]. However, for a sufficient prevention of the alumina condensation, we have chosen to mix the ionic aluminium precursor [Al(H_2_O)_6_]^3+^ with the PEO/EtOH/H_2_O solution under ice-cooled conditions before transferring the mixture into an oil-bath at 25 °C shortly before addition of the proton scavenger, propylene oxide, which will start the condensation and induce the phase separation.

**Scheme 1:**
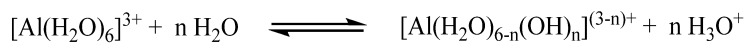
Hydrolysis reaction of aluminium precursor.

Upon introduction of the proton scavenger, the pH is increased, thus shifting the equilibrium of the hydrolysis reaction to the product side (Scheme 1). Via subsequent condensation (olation and/or oxolation) metastable oligomeric aluminium hydroxides and oxo-hydroxides are formed [28]. By further polycondensation of the oligomeric alumina species under controlled conditions, a phase transition from sol to gel can be achieved in a controlled environment (20 min in closed vessels at 40 °C).

**Figure 5:**
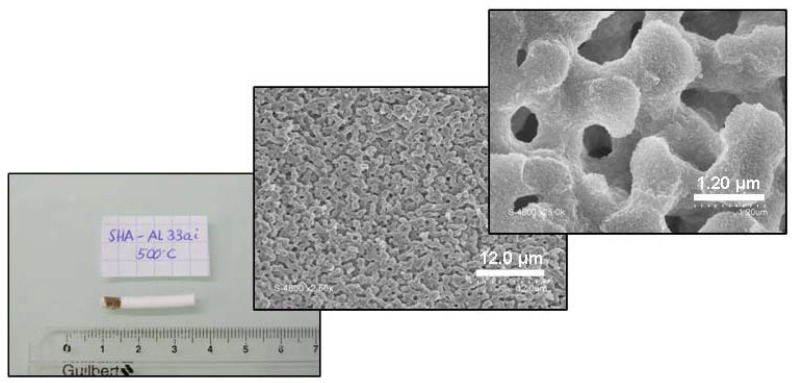
Photograph of the alumina monolith after calcination at 500 °C and the corresponding SEM pictures showing its isotropic and homogeneous macroporous network (macropores ~1 µm) and its skeleton (thickness ~1 µm).

The aim of the synthesis is the fabrication of hierarchically porous monoliths exhibiting a continuous macroporous morphology and a further porosity on a different length scale such as mesoporosity. Thus, a phase-separation inducing agent such as PEO, which is a highly hydrophilic polymer, is employed to generate a continuous morphology in the micrometer range. It is known from the synthesis of hierarchically organized SiO_2_ monoliths that with increasing size of the polymerizing SiO_2_ oligomers, the miscibility of the polar solvent-rich and the less polar polymer-rich phase (enhanced polycondensation of SiO_2_ oligomers due to agglomeration at the ethylene oxide units of the PEO via hydrogen bonding) is gradually reduced and phase separation occurs. This phase separation is then subsequently frozen by sol-gel transition. If the phase separation occurs via spinodal decomposition, the resulting morphology consists of bi-continuous domains with diffused interfaces, which only after further solidification of the silica network evolve sharp solid/liquid interfaces [31,32]. It is highly assumed that a similar mechanism also occurs for the alumina system [28,33].

**Figure 6:**
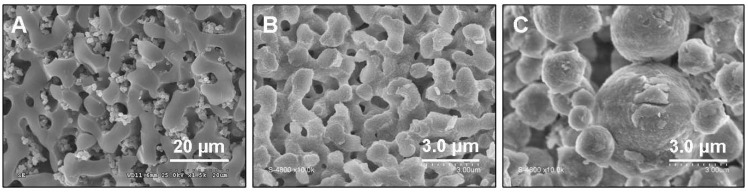
SEM images exhibiting the impact of the varying molar ratio of PEO (M_V_ = 10^6^) on the resulting morphologies in the micrometre range (**A**) Al/PEO = 1/3.9 × 10^−6^; (**B**) Al/PEO = 1/4.5 × 10^−6^; (**C**) Al/PEO = 1/5.1 × 10^−6^.

This synthesis is very sensitive and we show in Figure 6 that even slight changes in the molar ratio of Al/PEO have a major influence on the resulting macroporous morphology from bi-continuous to particulate as proven by the SEM images (Figure 6). A highly bi-continuous alumina skeleton was obtained with Al/PEO ratio to 1/4.5 × 10^−6^, whereas an agglomerate of particles was obtained for Al/PEO ratio to 1/5.1 × 10^−6^.

**Figure 7:**
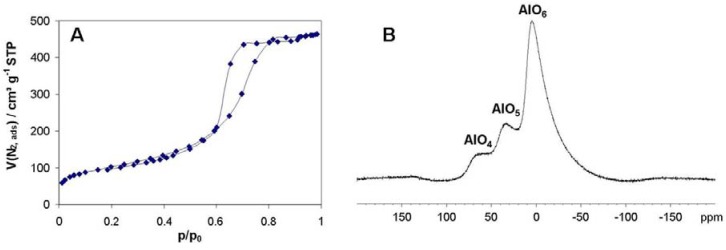
(**A**) Typical N_2_ sorption isotherm at 77 K for alumina monoliths and (**B**) ^27^Al MAS NMR spectra recorded at a steady spin rate of 10 kHz of an alumina monolith (Al/PEO = 1/5.1 × 10^−6^).

The corresponding nitrogen sorption isotherms (Figure 7A) of all alumina monoliths (Table 1) exhibit a high surface area (~400 m^2^ g^−1^), a large pore volume (0.7 mL g^−1^), a quite narrow mesopore size distribution (~7 nm) for the mesopore system resulting from interparticulate voids existing between the nanoparticles forming the monolith skeleton. It is known from XRD investigations that the alumina phase is completely amorphous after calcination of the monolith at 500 °C in air, exhibiting besides octahedral AlO_6_-sites also AlO_4_ and AlO_5_ (tetra- and pentahedral coordinated) species (Figure 2B).

The main drawback of this procedure is the difficulty to control the parameters of the synthesis, the large shrinkage of the monolith upon synthesis (from 8 mm in the mold to 3 mm after synthesis), and the high temperature needed to get the γ-Al_2_O_3_ phase (500 °C is not enough), but this represents the only example to synthesize an alumina monolith with hierarchical porosity suitable for continuous flow application [18].

**Table 1:** Textural properties of alumina monoliths obtained with different Al/PEO ratios.

	N_2_-sorption		
Al/PEO	S_BET_/m² g^−1^	D_BdB_/nm	V_p_/cm³ g^−1^
1/3.9 × 10^−6^	56	-	0.4
1/4.5 × 10^−6^	364	7.0	0.7
1/5.1 × 10^−6^	313	7.0	0.6

**Table 2:** Comparison of the textural properties of the mesoporous alumina (γ-Al_2_O_3_) prepared by different routes.

	N_2_-sorption		
γ-Al_2_O_3_	S_BET_/m² g^−1^	D_BdB_/nm	V_p_/cm³ g^−1^
Rapid and easy sol-gel	338	11.0	1.10
Nanostructured alumina (EISA)	197	8.6	0.38
Macro-/mesoporous Monolith	364	7.0	0.70

## 3. Experimental Section

### 3.1. Chemicals

*Block*-poly(ethylene oxide)-*block*-poly(propylene oxide)-*block*-poly(ethylene oxide) (P123, M_av_ = 5,800, EO_20_PO_70_EO_20_), poly(ethylene oxide) (PEO, M_av_ = 1,000,000), dodecylamine (DDA), 2-butoxyethanol, and (±)-propylene oxide (PO) were purchased from Sigma-Aldrich. The precursors AlCl_3_·6H_2_O, Al(O*sec*Bu)_3_, and Al(O*i*Pr)_3_ as well as all employed solvents (ethanol, butan-2-ol, *etc*.) and acids (HNO_3_, 68%) were purchased from Sigma-Aldrich and were used without further purification.

### 3.2. Materials Synthesis

#### 3.2.1. Nanostructured Mesoporous Amorphous Al_2_O_3_ Powders

In a modified synthesis based on the synthesis procedure presented by Yuan *et al*. [20] the precursor Al(O*i*Pr)_3_ was added dropwise to a pre-homogenized solution of P123 in EtOH and HNO_3_ under vigorous stirring at room temperature (RT). The molar ratio of the synthesis mixture was adjusted to 1:0.017:34.1:2.09 Al(O*i*Pr)_3_/P123/EtOH/HNO_3_. The sol was stirred for 5 h at RT before it was transferred to an oven with ventilation at 60 °C for 2 days to introduce the evaporation-induced self-assembly in order to obtain a 2D-hexagonally nanostructured mesoporous alumina network and for a subsequent drying of the final material. Without a further washing step, the incorporated polymer was eliminated by calcination at 400 °C in a tubular furnace under air flow with a heating ramp of 1 °C min^−1^ for 4 h.

#### 3.2.2. Disordered Mesoporous γ-Al_2_O_3_ Powders

Disordered mesoporous Al_2_O_3_ powders exhibiting high specific surface areas were synthesized by a new precipitation strategy in 2-butoxyethanol. In a beaker Al(O*sec*Bu)_3_ was combined with 2-butoxyethanol to prevent early hydrolysis and condensation of precursor. An aqueous solution of dodecylamine (DDA) in 2-butoxyethanol (2-BuOEt) was slowly added (within 20 min) under vigorous stirring at RT. During the addition of the aqueous phase, first a gel was formed which was then destroyed by further addition of the aqueous phase resulting in a precipitation of alumina. The molar ratio of the sol was 1:0.04:5.27:17.00 Al(O*sec*Bu)_3_/DDA/H_2_O/2-BuOEt. The resulting precipitate was aged for 24 h at 33 °C, filtrated and washed 3 times with *sec*BuOH at RT. The resulting alumina powders were first dried at 80 °C for 1 day before they were calcined at 500 °C for 5 h in air (heating ramp: 2 °C min^−1^).

#### 3.2.3. Hierarchically Macro-/Mesoporous Alumina Monoliths

Hierarchical alumina monoliths were prepared via a modified synthesis pathway based on the preparation given by Tokudome and Nakanishi [28,33] by employing AlCl_3_·6H_2_O as precursor. PEO (0.08 g) were first dissolved in 5.5 mL Ethanol and 4.0 mL H_2_O (58:42 v/v EtOH/H_2_O) at 0 °C. Only after complete dissolution of PEO, 4.32 g AlCl_3_·6H_2_O were added under vigorous stirring at 0 °C. For complete dissolution of the aluminium salt, the reaction mixture was then maintained at 25 °C in an oil-bath and 3.8 mL propylene oxide (PO) was quickly added. Within 3 minutes the pH of the sol raised from 1 to 3 and the sol was transferred to vessels. The vessels were sealed and the system was allowed to gel and age at 40 °C for 2 days. After 20 min at 40 °C, the sol-gel transition as well as a macroscopic phase separation could be observed, resulting in turbid gel bodies.

After aging, all gels were washed in 2-propanol at 60 °C (4× solvent exchange within 6 h) before they were slowly dried from RT to 40 °C within 7 days. The monoliths were calcined from RT to 500 °C with intermediate heating plateaus at 100 °C and 250 °C. Each temperature was held for 5 h and the heating ramp between the plateaus was 1 °C min^−1^.

### 3.3. Characterization

All samples were characterized by nitrogen sorption analyses. The adsorption-desorption isotherms of the calcined samples were measured on a Micromeritics ASAP 2010 device at 77 K. All samples were degassed at either 150 or 250 °C in vacuum for 8 h prior to the analysis. The specific surface area was determined by the BET method for adsorption data in the relative pressure range from 0.05 to 0.20 [34]. The pore volumes were calculated from the quantity of nitrogen adsorbed at a relative pressure of 0.97. Average pore diameters were determined from the nitrogen desorption branch according to Broekhoff and de Boer (BdB) [35,36].

Powder X-ray diffraction (XRD) patterns were recorded on a Bruker AXS D8 Diffractometer by using CuKα radiation and a Ni monochromator.

The macroporous morphology of the monoliths and the particle morphology were examined on a Hitachi S-4500 I Scanning Electron Microscope (SEM).

All ^27^Al Magic Angle Spinning NMR spectra were recorded on a Bruker DSX-300 spectrometer operating at 78.2 MHz and a spinning rate of 10 kHz. A single pulse sequence with a short pulse length of 0.5 µs was employed and a delay of 1.0 s allowed the complete relaxation. For one spectrum 1000 scans were accumulated.

## 4. Conclusions

Today a wide variety of different synthesis strategies are proposed to obtain mesoporous alumina materials. The synthesis has to be chosen depending on the required properties such as ordered mesoporosity, high specific surface areas and pore volumes, crystallinity, hierarchical structures, *etc.* In this paper we have presented the most convenient strategies in order to fabricate reproducible mesoporous alumina materials with tailored properties (summary in Table 2). For theoretical studies the use of hexagonal ordered alumina is the most suitable and this synthesis is reached using a triblock-copolymer surfactant in acidic media by the Evaporation Induced Self-Assembly. However, this synthesis is difficult to scale up for industrial applications as catalytic support. If large scale synthesis of mesoporous alumina with high specific surface areas and pore volumes is requested, the precipitation process in 2-butoxyethanol has proven to be a very convenient strategy with respect to reproducibility, cost-effectiveness, reaction time, energy input and prices for the educts. For the transfer from particulate to macro-/mesoporous monolithic alumina materials only few synthesis procedures are described in the literature until today. The procedure published by Nakanishi *et al*. [28,31,32,33] is the most promising. The bi-continuous macroporous morphology obtained in this preparation of alumina monoliths is proper for high mass transfer and the high specific surface area is suitable for high loadings of reactive catalytic species. These monoliths are therefore very attractive support materials for applications in continuous flow.
